# Cave Pearl Data Logger: A Flexible Arduino-Based Logging Platform for Long-Term Monitoring in Harsh Environments

**DOI:** 10.3390/s18020530

**Published:** 2018-02-09

**Authors:** Patricia A. Beddows, Edward K. Mallon

**Affiliations:** 1 Department of Earth & Planetary Sciences, Northwestern University, 2145 Sheridan Rd–Tech F374, Evanston, IL 60208-3130, USA; 2 Triple Point Design, LLC, Evanston, IL 60202-1125, USA; edward.mallon@gmail.com

**Keywords:** data logger, environmental monitoring, Arduino, open source, submersible, underwater, cave, Yucatan Peninsula, vadose hydrology, subterranean karst estuary

## Abstract

A low-cost data logging platform is presented that provides long-term operation in remote or submerged environments. Three premade “breakout boards” from the open-source Arduino ecosystem are assembled into the core of the data logger. Power optimization techniques are presented which extend the operational life of this module-based design to >1 year on three alkaline AA batteries. Robust underwater housings are constructed for these loggers using PVC fittings. Both the logging platform and the enclosures, are easy to build and modify without specialized tools or a significant background in electronics. This combination turns the Cave Pearl data logger into a generalized prototyping system and this design flexibility is demonstrated with two field studies recording drip rates in a cave and water flow in a flooded cave system. This paper describes a complete DIY solution, suitable for a wide range of challenging deployment conditions.

## 1. Introduction

The challenges of climate change, resource depletion and urbanization highlight the global need for environmental monitoring systems. The cost of commercial instruments is a significant barrier to establishing large monitoring networks, even for well-funded research programs. The goal of this open-source project is to develop a robust data logger prototyping system that is inexpensive enough to bring multi-unit sensor deployments within the range of limited research budgets and accessible to those with modest electronics experience.

Desirable characteristics for this monitoring platform include:

- Built from a small number of low-cost, readily available components
- Supports a wide variety of analog and digital sensors
- Non-proprietary software and file formats
- Removable microSD storage media
- User-adjustable operating parameters, such as sampling interval
- Operating life span > 1 year using alkaline AA batteries transportable on most airlines
- Rugged, chemically resistant environmental housing for submerged or buried deployments

Instruments that monitor aquatic and subterranean environments are among the most expensive commercial sensors available, so several groups are working to create less expensive alternatives with Arduino microcontroller boards. The Arduino platform combines electronics hardware and software into a cohesive system that is easy for novices to use in many different applications including lab and field based research [1]. One great strength of the Arduino is the cross-platform Integrated Development Environment (IDE) which presents a simplified C++ programming interface that leverages extensive code libraries without requiring the user to know low-level details for common-case implementations [2,3,4].

The citizen-science movement is using this open-source tool at community-based organizations like PUBLIC Lab [5], which has several ongoing Arduino-based water quality monitoring projects including the KnowFlow [6] and the Riffle [7]. Generally speaking, instruments from projects at the community level are not yet capable of multi-month marine deployments.

Academic groups studying subaquatic environments are also building instruments with this open-source hardware. Notable examples include: the surface drifter, which measures multiple parameters including conductivity, temperature and GPS position [8]; the kDuino Buoy, which measures water transparency by deriving diffuse attenuation coefficients from inexpensive light-to-frequency sensors [9]; the in-situ fluorescence monitor, which uses LEDs to excite chlorophyll *a* and measures the resulting fluorescence with a silicon photodiode [10]. These projects achieve measurement quality comparable to commercially available sensors costing many thousands of dollars. However, even with large capacity batteries, the instruments deliver operating times less than one month, because they use large form-factor Arduinos and plug-in data logging shields with relatively high power consumption. In addition, many projects use readily available housings with IP68 ratings that limit these instruments to shallow deployments (e.g., the Otterboxes used in [10,11]). Consequently, many groups including this project, are experimenting with DIY housings constructed from readily available PVC tubing (see also [8,12,13]).

The proliferation of alternative Arduino-compatible boards is providing a range of new hardware options with significantly better power performance in logging applications. Unfortunately, many projects that take advantage of these small form factor boards rely on customized carriers that significantly increase the cost of the final assembly [11,13]. The pursuit of further optimization has led some open-source projects to abandon Arduino style boards and create custom printed circuit board (PCB) solutions around the 328P microprocessor [7,14]. The use of bespoke PCB options places these devices outside the reach of users with limited electronics experience.

The logger described in this paper provides >1 year run-times and excellent environmental durability, while maintaining accessibility. A key feature of this “breakout modules and jumper wires” approach is that the core components are all replaceable by alternate parts from the Arduino ecosystem without requiring significant changes to the operating software. No additional PCB’s are required and open source libraries facilitate the addition of different sensor modules to this data logger with minimal programing skills. The Cave Pearl Project has also benefited by from feedback in a classroom setting where college undergraduates assemble a breadboard version of the logger and proceed to building a field deployable unit in an introductory level course on environmental monitoring [15,16].

This paper begins with an overview of the physical construction of the logger platform, the PVC housings and important factors when selecting sensors. The data logger software is then presented, which uses character arrays in generic data handling functions that novice programmers can understand and modify. Power-optimization of the loggers is then discussed with a prioritized list of software and hardware techniques that collectively extend the run-time well past one year of operation. Two case studies demonstrate the versatility of this data logger for environmental monitoring, showcasing variants that use similar accelerometer sensors in different physical housings to log drip events (Case Study 1, Section 7) and to monitor water flow. (Case Study 2, Section 8). The housings developed by the project can withstand submersion at depth for extended periods and are a significant innovation that enables users to reconfigure both the enclosure and the electronics to suit specific research questions.

## 2. Data Logging Platform and Principles of Operation

**Figure 1:**
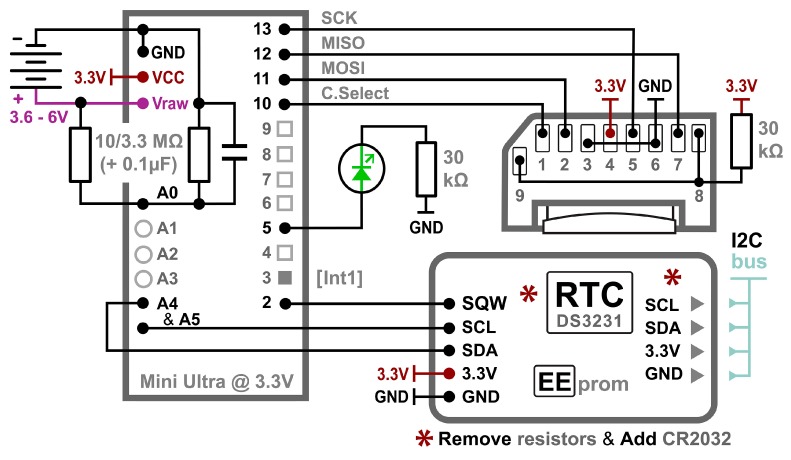
Arduino-based data logging platform connection plan, shown with the pin layout of Rocket Scream Mini Ultra, a DS3231 Real Time Clock (RTC) module with an onboard 4 kB EEPROM and a Raspberry Pi microSD card adaptor board. Unused connections **8** and **9** on the SD adapter are pulled up to prevent power loss due to floating inputs. At least one indicator LED is recommended, shown here with a 30 kΩ limiting resistor. This basic connection plan can be adapted to any 3.3 V Arduino compatible board by moving the jumpers to accommodate the physical pin locations on modules from different vendors.

The data logger itself has three primary components: a microcontroller board, a real-time clock (RTC) module with EEPROM memory and a microSD card adaptor (Figure 1). Small form-factor 3.3 V Arduinos are used because they allow SD memory to be connected to the serial peripheral interface (SPI) input/output (I/O) pins without logic level shifters. Most modern integrated circuit (IC) sensors can also be connected directly to boards with 3.3 V logic levels. These “mini-Arduinos” are programed using a separate Universal Asynchronous Receiver/Transmitter (UART) adapter, which is advantageous in a logging application because removing the UART chip conserves power and reduces the physical size of the controller board.

Many Arduinos are based on Atmel 8-bit RISC microcontrollers—collectively called AVRs—that provide several memory, speed and input/output (I/O) options [17]. AVR’s with the pico-power (P) designation offer advanced power saving options, with the deepest processor sleep states drawing as little as 1.7 µA [18]. The IDE embeds support for these AVR chips when programs are compiled, allowing the majority of Arduino compatible boards to run the same code.

**Figure 2:**
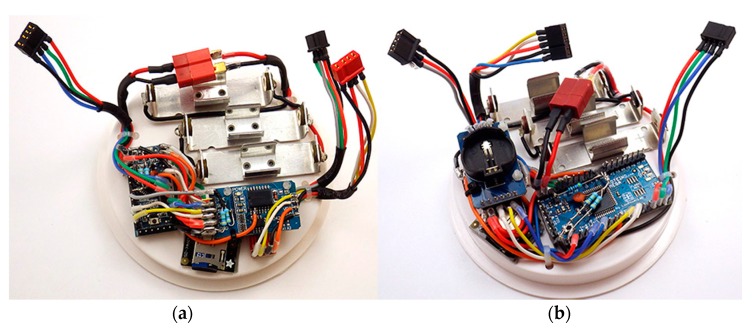
Data loggers following the connection plan outlined in Figure 1, assembled on 4 inch knockout test caps. (**a**) Rocket Ultra board using the 328P ATmega chip, by Rocket Scream. (**b**) Moteino MEGA board using the 1284P ATmega chip, by LowPowerLab. Both data loggers have the I2C bus broken out with 4-pin Dean’s micro plugs for external sensor connections and include additional connectors for a 3-color LED and for 1-wire bus sensors. (Note: The 1-wire bus is not shown in Figure 1).

This family of code-compatible AVR processors expands the range of prototyping projects that can be built with the basic logger plan. For example: two readily available Arduino compatible boards are the 328P based Ultra by Rocket Scream (Figure 2a) and the 1284P based Moteino MEGA by LowPowerLab (Figure 2b). The data logger’s code can be compiled for either board by selecting the appropriate board definition in the IDE. The 1284P is a faster processor, enabling rapid sampling with circular buffering strategies that would exceed the SRAM available on 328P based boards. The trade-off is that the 1284P uses 3× more power during run-time operations. Both processors support the low current sleep modes required for data logging applications.

The Arduino’s 10-bit single-ended analog-to-digital converter (ADC) provides 1024 sampling levels between a common ground and the 3.3 V positive rail. The default resolution is 3.22 mV/bit (i.e., 3.3 V Aref/1024 bits); however, increased resolution is possible using the internal 1.1 V band-gap reference on AVR processors. Distributing those ADC levels over a reduced input range from 0–1.1 V resolves 1.074 mV per bit. The analog A0 input pin in this logger plan is used to monitor the main battery via a 3.3 MΩ/10 MΩ voltage divider which reduces the raw battery voltage to 25% of actual so that it falls within the ADC’s input range. Large value resistors are used to limit power loss, so a small 1µF capacitor is added on the ground side of the divider to enable reading by the ADC; which normally requires an input impedance below 20 kΩ. Pins A4 and A5 are reserved for inter-integrated circuit (I2C) bus communications with the RTC and various sensor modules. On most 328P based boards, this leaves 5 remaining pins available for use with analog sensors (A1–A3, A6 and A7).

Most 328P-based Arduinos have 13 digital I/O pins, of which 7 are used in this data logger configuration (Figure 1). Pins D0 and D1 are typically reserved for serial outputs through a UART/USB adapter because after execution of a **Serial.begin()** command during debugging, those pins cannot be used for other digital inputs. Hardware interrupt pin D2 is connected to the RTC’s programmable alarm to wake the processor from low-power sleep modes. Pin D3 provides a second hardware interrupt line that can be triggered in a similar manner by sensors. The indicator LED is on pin D5. Four digital pins are used for SPI communications with the microSD card: D10 as a Cable Select (CS) line to avoid communication conflicts with other SPI devices, D11 for Master-Out-Slave-In (MOSI), D12 for Master-In-Slave-Out (MISO) and D13 for the bus clock line (SCL). This configuration leaves 6 digital pins available for sensor input (D3-if not used as an interrupt, D4 and D6–D9). Analog inputs A1–A3 can also reprogrammed to act as digital I/O ports if needed.

A high-accuracy Real Time Clock (RTC) is required for environmental monitoring applications as the oscillator circuits on most Arduino boards have significant variation due to thermal effects. The DS3231 RTC module used in this logger (visible in Figure 2a,b) includes a temperature-compensated crystal oscillator with ±1 min drift per year and the 0.25 °C resolution temperature readings used for that correction can be read from the RTC’s data registers [19]. The module also includes a 4 kB AT24C32 EEPROM [20] and a bank of 4.7 kΩ resistors which pull-up the I2C bus lines before they pass to a cascade port where additional sensors can be connected. The DS3231/AT24C32 module described here has an inefficient trickle charging circuit to maintain an LIR2032 backup battery. This circuit does not function properly at 3.3 V and should be disabled by removing the associated 200 kΩ resistor from the module (Figure 1 and Section 6.1). At that point, a non-rechargeable CR2032 coin cell should be used, which will provide backup power for >5 years of RTC operation.

Cave Pearl loggers store time-stamped sensor readings in CSV format on microSD cards using Greiman’s SdFat library [21]. This allows up to 4 GB of storage and data retrieval without proprietary software. This ease-of-use increases power requirements as SD cards can draw up to 200 mA during normal file opening, saving and closing procedures. Unpredictable factors like the data-writing sequence or age-related wear-leveling can occasionally keep cards drawing power for 10’s to 100’s of milliseconds beyond their normal duty-cycle. The frequency of these high-power drain events is reduced significantly by buffering sensor readings in the EEPROM on the RTC module before transferring that accumulated data to the SD card in a batch process. A typical logger deployment with a 15-min sampling interval generates less than 5 MB of CSV format data over one year, so smaller 256 MB to 1 GB SD cards are sufficient. Most SD cards automatically enter a low-power mode when no SPI bus clock is applied but some cards enter standby states more quickly if pullup resistors are enabled on the MOSI and MISO lines. The 328P’s internal pull-up resistors can be used for this and to pull up the CS line.

Power failures during SD save events can damage data files on the card. To prevent this, the main battery voltage is checked by reading the divider on pin A0 before any data is transferred. The battery is checked again after the file is closed and the logger automatically goes into a controlled shut-down if either reading approaches the minimum input voltage required by the 3.3 V regulator. This cutoff is set at 3.65 V in the provided code [22], which can be modified to support regulators with different drop-out ratings.

**Figure 3:**
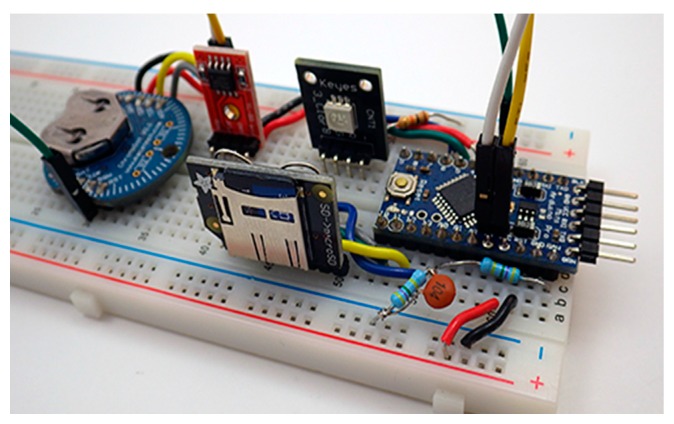
A typical breadboard testing setup to assess sleep current and verify code operation prior to soldering. The configuration shown includes a no-name clone of the Sparkfun Pro-Mini, a Macetech Chronodot DS3231 RTC, a stand-alone AT24C512 EEPROM module, a KEYES 5050 LED module and an Adafruit micro SD card adapter. Typical sleep currents at 3.3 V for the main components are: DS3231 module (0.08–0.09 mA), Sleeping micro SD card (0.05–0.09 mA), Pro Mini style board (0.02–0.06 mA).

This modular build plan addresses the constantly changing part availability in the hobbyist market because a logger can be assembled from many different breakout boards. The breadboard arrangement shown in Figure 3 is functionally identical to the logger shown in Figure 2a but it uses a different set of components. The same code functions on both builds with no changes but good working practice requires testing of each unique combination to verify that selected parts do not consume excessive amounts of power. A breadboard test is also helpful when debugging code for integrated chip based sensor modules which can vary significantly between vendors. 

**Figure 4:**
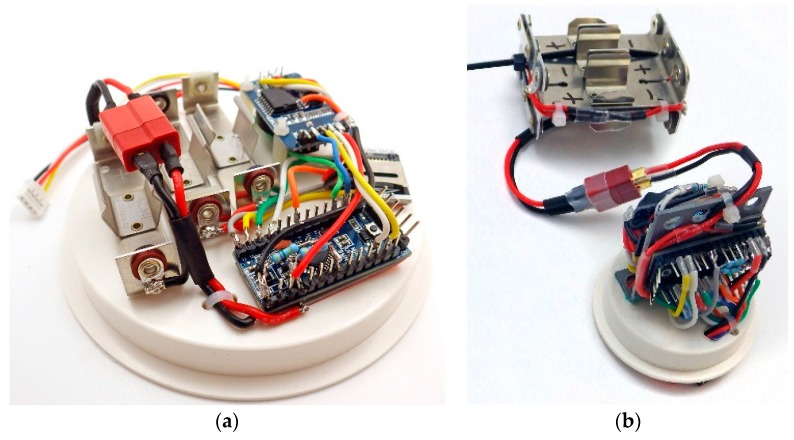
Alternative physical arrangements of the same electronic components: (**a**) Surface logger configuration: components are mounted on a 4 inch ABS knockout cap with double sided tape. The RTC board is supported by 12 mm M2 nylon standoffs. (**b**) Submersible logger configuration: components are re-arranged to fit inside a 2 inch diameter pipe housing. Double-sided tape attaches the modules to an L bracket made from thin ABS plastic that is solvent welded to a 2 inch knockout cap. Wires pass through holes in the bracket to connect components on opposite sides. The battery pack shown has two banks of 3 × AA batteries in series, which are isolated from each other with Shottky diodes.

Once module compatibility has been confirmed, the components can be soldered into a permanent configuration. The physical arrangement of the component modules can be changed to accommodate the different housings for deployments above and below the water table (Figure 4a,b). A completed logger typically has a materials cost between $ 25–50 before sensor modules are added (Appendix A Table A1). Assembly time is 5–10 h depending on the complexity of the housing (and complete curing of the potting epoxy requires 2–3 days). Detailed assembly instructions for a 4 inch surface logger platform are posted—and regularly updated—on the Cave Pearl Project blog [15].

## 3. Environmental Housings

In keeping with the DIY ethos of the project, the authors created and tested several housing configurations made from common Poly Vinyl Chloride (PVC) fittings. PVC is waterproof and chemically resistant but it degrades when exposed to UV so a protective coating of water based exterior latex paint is advisable for surface exposures. Deployments protected from direct sunlight are recommended to reduce thermal stress on the electronic components.

**Figure 5:**
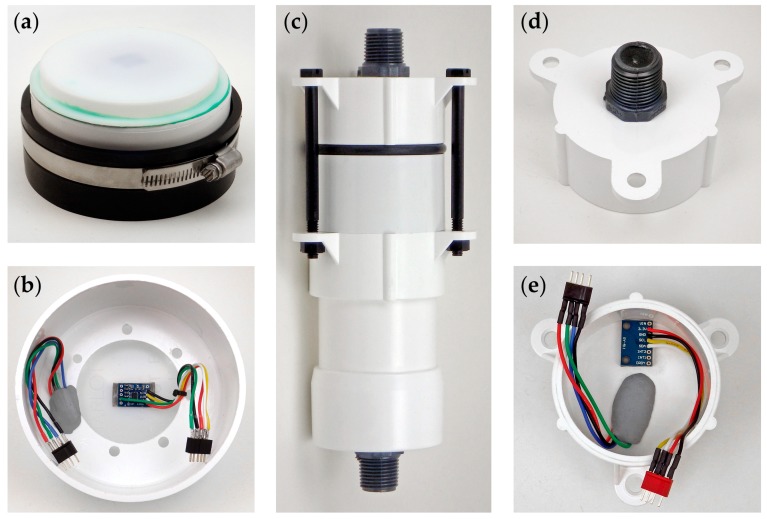
Environmental housings for Cave Pearl loggers. Surface logger: (**a**) Drip-sensor unit with a “Charlotte” brand translucent knock-out plug, which is solvent welded to the top of a 4 inch PVC end cap. A flexible rubber end-cap with a standard pipe clamp completes the housing with a water-tight seal. (**b**) Inside-view of the drip-sensor lid, showing a circular cutout of the original PVC end cap with the accelerometer mounted on the inside of the knock-out plate. A tri-color LED is mounted on the PVC so that it is visible through the translucent surface. Submersible logger: (**c**) Complete submersible housing made from 2 inch pipe fittings. Nylon bolts hold the body together, compressing the central O-ring seal. (**d**) Outside view of the submersible housing end cap. An RGB indicator LED is potted with transparent epoxy inside the grey ½ inch threaded connector. (**e**) Inside view of the sensor cap showing the plumber’s putty used to plug the housing penetration so that liquid epoxy could be poured around the LED. WS Deans’ 1241 micro connectors link wires from the sensor caps to the logger platform.

Surface housings consist of a black flexible test cap held onto a 4 inch diameter PVC drain cap with a stainless-steel pipe clamp (Figure 5a). Sensors can be potted in epoxy wells on the PVC lid following the detailed instructions on the Cave Pearl Project blog [23]. For the drip-event counter in Case Study 1 (Section 7), a task-specific variant of this housing was created with a translucent ABS knockout plate solvent welded to the drain cap and an accelerometer attached to the inside surface with double sided tape (Figure 5b). Vibration from a drop that impacts this thin ABS plate triggers a tap sensing alarm on the accelerometer, which increments an interrupt-driven counter. The status indicator LED is visible through the translucent knockout plate, eliminating housing penetrations. This housing has survived >3 years of continuous operation during field tests in a >90% relative humidity cave environment (see Section 7.4).

**Figure A1:**
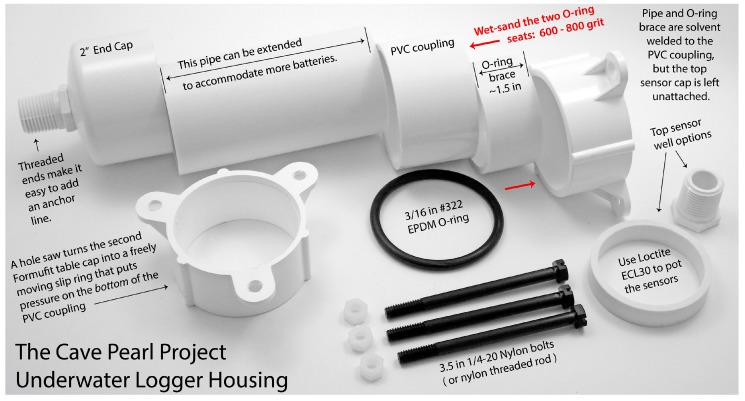
Exploded view of the 2 inch pipe submersible housing components and their order of assembly. The casting seams on the two indicated surfaces need to be sanded smooth for a proper O-ring seal. Proper cleaning and priming procedures should be followed to assure strong joins and heavy duty PVC cement should be used for maximum bond strength. Options chosen for potting the sensors should be deep enough for 8–10 mm of epoxy over all components not designed for high pressure applications. The 1/4–20 nylon bolts shown vary slightly in diameter from one supplier to the next and sometimes the holes in the struts need to be slightly enlarged by drilled out to let the upper cap slide freely. Some PVC couplings may need slight trimming to an optimal length of 1.5 inches.

The housing for underwater deployments is constructed from a 2 inch schedule-40 PVC pipe. (Figure 5c). Three 1/4–20 nylon hex bolts are threaded through Formufit table leg end caps [24] to compress an EPDM 332 O-ring. The O-ring seats are wet-sanded to 800 grit and the housing has proven robust on deployments to 24 m for >2 years. The upper cap slides freely, increasing the strength of the O-ring seal with depth. This metal-free design is impervious to salt water corrosion; however, the nylon bolts expand ~2 mm in length during extended marine deployments. This is mitigated by pre-soaking the bolts for one week prior to deployment. The component list and prices—as of January 2018—are listed in Appendix A Table A1 and an exploded assembly figure for the underwater housing can be found in Appendix A Figure A1.

Moisture resistance is critical when selecting potting compounds and some epoxies degrade rapidly when exposed to marine salinity. Modules embedded in Loctite E-30CL epoxy [25] have proven durable over >2 years on ocean deployments. For deeper deployments, ~8–10 mm of epoxy should overlay any components that are not designed for high pressure environments. Epoxy will bow under pressure and this led to the failure of some sensor ICs that were mounted under relatively thin layers of epoxy when they were deployed at depths below 20 m.

By the end of 2017, more than 30 flow sensors have been constructed with the 2 inch housing shown in Figure 5c and additional loggers have been constructed with other sensor configurations. Considering only the flow monitoring units described in Case Study 2 (Section 8), the project has accumulated 261 months of underwater operation over 42 deployments. Including the submerged units logging pressure and temperature, the total is 430 months of underwater operation over 78 deployments. The average deployment time is 5 months and the longest continuous deployment of this housing style is 16 months. These deployments span a range of depths from 2 to 24 m. Housings constructed in 2015 are still in service and none of the 2 inch housings have leaked to date.

The small physical profile of these underwater housings facilitate deployment by a diver. SCUBA training organizations recommend dive depths of no more than 40 m (130 ft.) because of the risk of narcosis and pressure at that depth is less than 60 psi. Two inch schedule 40 PVC pipe has a rated operating pressure >150 psi and a collapse pressure of >225 psi [26,27]. These underwater housings easily withstand the full depth range accessible to research SCUBA divers breathing air.

## 4. Sensors, Communication Protocols & Resolution

Digital sensor data is often delivered with one of three common bus protocols: Inter-Integrated Circuit (I2C), Serial Peripheral Interface (SPI) and one-wire. SPI and I2C are supported natively by hardware inside the AVR processor and software libraries are available to support other communication protocols such as 1-wire. Many digital sensors include high bit-depth ADCs and the programmable threshold alarms available on some sensors make it possible to capture event-driven phenomenon, which would be difficult with analog sensors.

I2C is a mature communication protocol that is used to attach relatively low speed sensors to microcontrollers over short distances (typically less than 1 m). The wide variety of sensors and ready availability of open-source code libraries make this protocol the best choice for Arduino based data loggers. I2C sensors can have sleep currents as low as 1 μA. The use of four-pin connectors with this parallel bus topology allows sensor caps (Figure 5) to be swapped between loggers, which is particularly useful for upgrades and repairs in the field. The I2C protocol supports more than a hundred devices on the bus, making it easy to build multi-sensor combinations with a range of resolution and accuracy specifications. Sensor breakout boards often come with surface mounted pullup resistors, which are redundant in this logger configuration because the data and clock lines are already connected to 4.7 kΩ pullups on the RTC module. For units with more than three sensors, some of those redundant pullup resistors may need to be removed so that the resulting parallel resistance does not fall low enough to interfere with bus communication.

SPI is the protocol of choice for storage and network applications where fast point-to-point communication is needed to move large amounts of information. However, SPI suffers from pin limitations, since each device needs a dedicated cable select line. In addition, SPI is a flexible standard with four different operating “modes”, with different clock polarity and phase with respect to the data. Arduino SD card libraries operate in Mode0 and adding a sensor to this logger which changes the SPI bus to a different operating mode would prevent data from being saved until the bus parameters are reset to Mode0 [28].

Sensors using the 1-wire protocol are ideal for applications requiring very long cable runs. This project has successfully deployed chains of twenty-four DS18B20 1-wire temperature sensors connected to Cave Pearl loggers with 30 m wires. The 1-wire protocol is proprietary to Maxim Integrated, so there are fewer 1-wire sensors available on the market compared to the range of options using SPI or I2C.

The project website provides tutorials to help people select an appropriate sensor to use with this data logger design [29].

## 5. Data Logger Software

**Figure 6:**
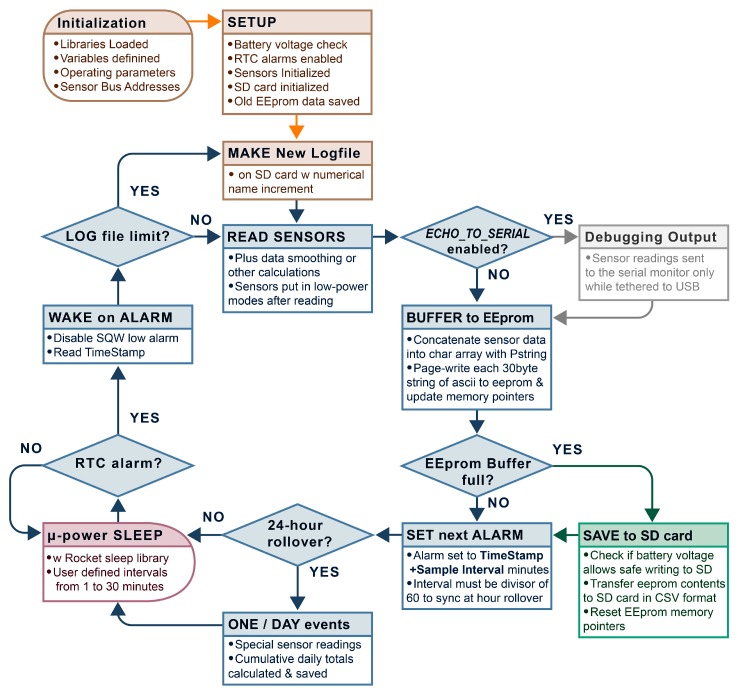
Flow chart of the Cave Pearl data logger software operation.

The logger base code provided on GitHub [22] is configured for the ADXL drip-sensor configuration described in Case Study 1 but that can be modified to support other sensors. At startup, attached sensors and the SD card are initialized. Then the entire contents of the EEPROM on the RTC module are transferred into a file on the SD card called EpBufer.csv so that no data from a previous logging run is accidentally overwritten. When those startup tasks are complete, the program enters a repeating loop of **sample → store → sleep → wake** which is the typical pattern for environmental monitoring applications (Figure 6). RTC alarms wake the sleeping data logger at set intervals to trigger each cycle and this continues until the main battery falls below 3.65 V, which automatically shuts down the data logger. 

Most logging platforms handle sensor data with highly-structured variables that reduce memory requirements and contribute to better power-management. However, these low-level formats also require custom software to convert the data into a human readable form. Instead, the Cave Pearl data loggers use a Print-to-String (PString) library to concatenate sensor readings into an ASCII character array. PString is a print-derivative library written by Mikal Hart that renders text from all of the variable types supported by the Arduino [30]. This unorthodox approach allows the primary data handling functions in the data logger’s base code to be completely generalized, thus accommodating sensor readings in any format. This library also prevents memory overflow errors, as PString simply ignores any excess data once the destination array is full. Even novice programmers can add new sensors to the Cave Pearl data loggers simply by including the matching sensor support library and altering straight forward statements (e.g., **str.print(***SensorReadingVariableName***)**). The char array that receives converted sensor readings is sized so that it can be transferred to the EEPROM with a single 32-byte page-write operation and this process is repeated until all the data from the current sensor reading cycle is stored.

When the memory address pointers indicate that the EEPROM storage is full, a function is triggered which transfers all the data from the chip to the micro SD card. The pointers are then reset and the next alarm time is programmed into the RTC based on the user-set sampling interval. The data logger then returns to a low-power sleep state until that alarm is triggered.

Saving the logs as CSV formatted text allows units to be serviced in the field by simply by replacing batteries and microSD cards. Turn-around time is an important consideration for field logistics in remote locations and slow downloads are particularly problematic in wet environments. These practical considerations outweigh code-level optimizations that require additional hardware or software to access the data.

## 6. Power Considerations for Maximum Run-Time

Adopters of this platform will use sensor combinations that address their particular research questions, resulting in unique duty cycles. Furthermore, each logger’s baseline performance is necessarily dependent on the specific components used in a build. This section uses conservative numbers drawn from typical part combinations and many builders will see significantly better performance with low-current sensors.

The drip counter unit example discussed in this section illustrates the relative contribution of the power optimization techniques in extending the operating lifespan. None of these techniques are proscriptive and the basic three component data logger is functional no matter which techniques the builder decides to implement.

**Figure 7:**
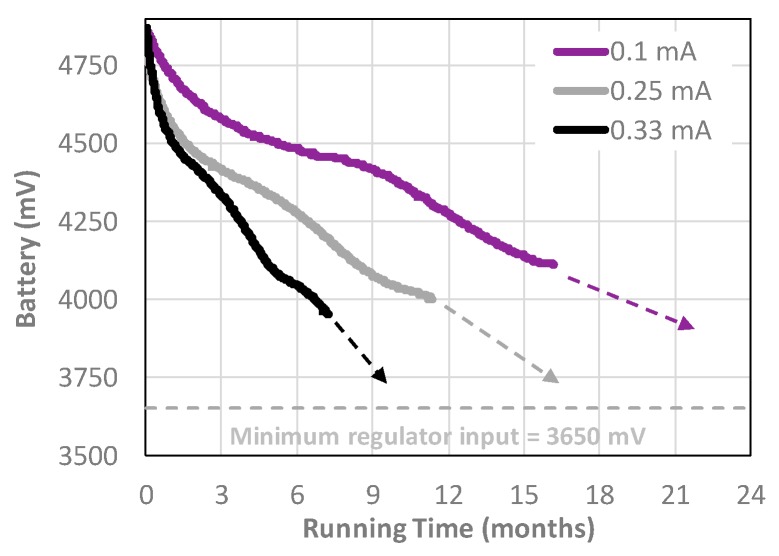
Data logger battery discharge curves for units with similar hardware powered by 3 × 1.5 V alkaline batteries in series. The 0.33 mA data logger built in early 2014 slept between readings but had no other power management. The 0.25 mA data logger used the described code-side techniques but had no hardware power optimization. The 0.1 mA data logger had software and hardware power optimizations, including an MCP1700 regulator, pin-powering of the RTC, a low current MS5803 pressure sensor and an SD card selected for its low-power sleep state. The dashed arrows indicate the estimated run-time to reach the 3.65 V shut-down limit.

The power consumed by the logger between sensor readings is the single most important factor determining a data logger’s operational lifespan. A data logger that draws a 0.25 mA sleep current requires ~21,600 mAs per day, even if the data logger does not wake up to take a reading. Given the effective yield from alkaline AA cells, a conservative operational lifespan at that sleep current is ~333 days. This is based on the typical yield from a battery pack with three AA alkaline cells in series, providing about 2000 milliampere-hours (mAh), though the rated capacity of the cells is often higher [31]. 2000 mAh × 3600 s/h = 7,200,000 milliampere-seconds (mAs). This is not a strict measurement of delivery potential because the voltage changes over time. It is however sufficient for the estimations done here. Cave Pearl data loggers at 0.25 mA sleep current have run longer than a year on real world deployments (Figure 7).

### 6.1. Optimizing Sleep Current

The Cave Pearl project uses several techniques (Table 1) to minimize sleep current and extend data logger operation time.

This project uses Atmel 328P based Arduino’s for the pico-power modes which can bring the processor down to a few µA while sleeping. It is easy to put the data logger into these sleep states using the Low Power library from Rocket Scream [32].

Power indicator LEDs on the Arduino board (and on digital sensor breakout boards) can draw more than 5 mA each, which is more than the microprocessors operating current. These are disabled by removing the limit resistor associated with each LED.

Sensor modules should operate natively at 3.3 V so that they can connect directly to the power rail on the Arduino, thereby avoiding additional regulators and the associated power losses. Selected sensors should go into low-current standby states automatically or have sleep modes that can be entered by setting control registers with bus commands. For example, the MS5803 pressure sensor from Measurement Specialties has a standby current of only 1 µA and the sensor does not require any specific commands from the logger to enter this state. Information about these sleep modes can be found on manufacturer data sheets.

**Figure 8:**
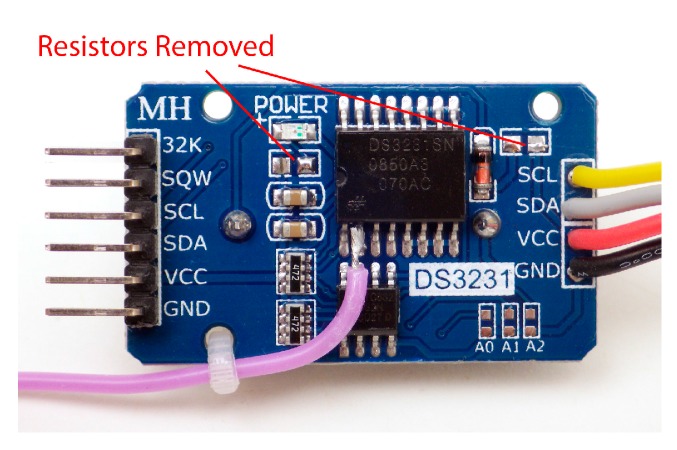
The power-input leg of the DS3231 IC is disconnected from the board and soldered to a wire (purple) for connection to a digital pin on the Arduino. When powered by the VCC line on the module, the RTC is responsible for almost 50% of sleep current of the entire data logger. This technique requires advanced soldering skills but pin-powering the clock IC can reduce a data logger’s sleep current to ~0.1 mA, allowing for operation >2 years on 3 × 1.5 V AA cells in series.

Some sensors can be powered by the Arduino’s digital I/O pins in OUTPUT mode, provided their maximum draw stays below the 25 mA pin limit. The DS3231 RTC is an excellent candidate for this technique, as it draws a constant 0.09 mA when powered by the VCC line on the module but only 3 μA when running from a backup coin cell. The power leg of the chip is lifted from the breakout board and wired directly to a digital I/O pin (Figure 8). Setting that pin high when the logger is awake powers the RTC chip during I2C communications. Driving the pin low forces the RTC to draw from the backup coin cell in a low-power timekeeping mode but the RTC can still generate alarms to wake the sleeping data logger. A 220 mAh CR2032 coin cell can power the DS3231 in this mode for >5 years. 

Sleep current can sometimes be improved by disconnecting sensors/devices using an NPN transistor as a ground-side switch controlled by a digital I/O pin. The OSBSS project [33] uses this technique to remove power to the SD cards, bringing sleep current into the 0.1 mA range for a Pro Mini based logger configuration similar to the Cave Pearl. However, SD support libraries for Arduino have no explicit support for multiple card re-initialization and that paper warns about ‘issues’ arising from frequent power-cycling of microSD cards. Power surges can cause problems with sensor reading stability and initialization delays make capturing short duration phenomenon challenging. The drip-sensors in Case Study 1 use a threshold alarm from an accelerometer (see Section 7.2), which requires that sensor to remain powered at all times. 

Discrete components such as voltage regulators, can have a significant impact on the overall power consumption of a breakout board even when otherwise identical sensors or processors are present. A logger platform based on a standard Pro Mini style board using the MIC5205 regulator may draw ~0.25 mA in sleep mode, while an otherwise identical build using a compatible board with the more efficient MCP1700 regulator would draw ~0.21 mA because of that regulators higher efficiency.

### 6.2. Optimizing Run-Time Power

Minimizing sleep current is critical for installations capturing time-series data at min-to-hours sampling intervals. For monitoring situations that require temporal resolution on the order of seconds, the power consumed during run-time events can become an equally important factor determining a loggers operating life (see Section 6.3). Table 2 provides a prioritized list of techniques that can reduce run-time current.

Many digital sensors offer programmable resolutions that alter their internal duty-cycle. For example, the common DS18B20 temperature sensor yields 9-bit data in 93 ms but can take up to 750 ms to deliver a 12-bit reading [34]. With this amount of variation, a good way to reduce run-time power is to put the Arduino’s processor to sleep while it is waiting for new data. A 15 ms delay at 5 mA uses 0.075 mAs but sleeping at 0.25 mA for that same interval requires only 0.0038 mAs. These millisecond-duration sleeps are also supported by the sleep library from Rocket Scream [32]. 

Sensor communication time is reduced by increasing bus clock rates from the conservative default settings. The I2C clock on an 8 kHz Arduino starts at 100 kbps but it can be accelerated to 200 kpbs by changing the Two Wire bit rate register (by assigning **TWBR = 12**) and increased to ~400 kpbs (by assigning **TWBR = 2**)*.*

Sensor selection should in part be based on power requirements during normal operation. The drip-sensors (see Section 7.2) use an ADXL345 accelerometer that draws a continuous 60 µA to provide processor interrupts. Comparable accelerometers are now available which use <10 µA in the same operating mode. 

For status indicators, green LED’s typically have 3 to 4 times the luminosity of other colors for the same power consumption. This allows the use of large 30 kΩ limit resistors and short 15 ms status blinks which have little impact on the power budget.

The power reduction register (PRR) can disable unused peripherals inside the AVR microcontroller chip. If you are not taking readings with the built-in ADC, turning off the ADC (by adding **ADCSRA = 0**; followed by **power_adc_disable()**; to the code) will save 0.23 mA of run-time current. Timer1 and Timer2 each draw 0.12 mA and these can also be disabled unless you are using pulse-width-modulated outputs. None of the PRR controlled sub-systems consume much power individually but turning them all off can reduce the running current on a 3.3 V Arduino by ~1 mA [35]. Do not disable Timer0, as this affects **delay()**, **millis()** and **micros()** functions within the chip. The Cave Pearl Data logger code uses the **micros()** function to track cumulative processor runtime.

### 6.3. Comparison of an Event Logger Duty-Cycle, without and with Run-Time Power Optimization

**Figure 9:**
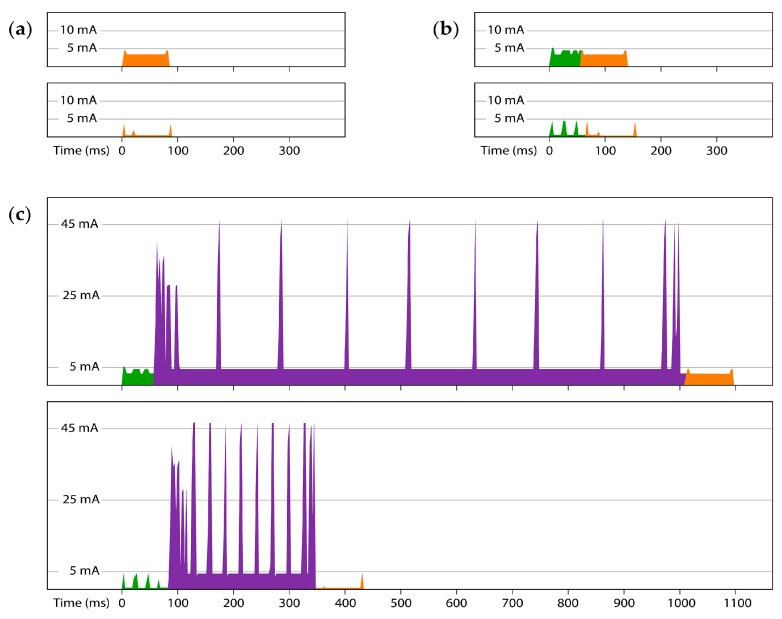
Current draw from a 4.6 V supply during the three events in the drip counter’s duty-cycle: (**a**) Drip counting (**b**) Reading sensors and buffering data to EEPROM (**c**) Transferring that buffered data to a no-name 1 GB microSD card. The upper graph of each pair shows current before run-time power optimization, while the lower graph in each set shows the same event after optimization techniques are applied. These records were captured with an Arduino UNO measuring the voltage drop across a 12 Ω shunt resistor at ~89 kHz (ADC prescalar = 8) [37].

The duty-cycle of an event counter, such as the drip sensor described in Case Study 1, consists of three events:

(a) Sensing an event: triggered when a drip impacts the housing (Figure 9a). An intermediate delay is required for damping of the vibrations before the movement threshold alarm can be re-enabled;
(b) Time Series event: At the end of each sampling interval the RTC alarm triggers a reading of the ambient temperature and writes that with the time-stamped count data to the EEPROM (Figure 9b);
(c) Transferring all buffered data from the EEPROM to the SD card, which occurs when the memory chip on the RTC module is full (Figure 9c).

This sequence is typical for loggers that count discrete events, like those connected to reed switch based tipping bucket rain-gauges and anemometers. 

The nested structure of the logger’s code means that the three duty-cycle events are contained inside each other, from smallest to largest. On Figure 9, the drip-counting event (orange) is encompassed by the RTC triggered data buffering event (green) and the SD saving event (purple) starts with the RTC sequence (green), then writes data to the SD card and then ends by passing through the drip counter (orange) vibration delay before the logger returns to sleep. 

Figure 9 and Table 3 show the current drawn during those duty-cycle events with two versions of the logger code running on the same data logger. The first plot in each set is not run-time power optimized and the second version includes the following power reduction techniques:

(a) Delay statements were replaced with brief processor sleeps
(b) PRR settings disable all internal peripherals except Timer0
(c) The I2C bus clock was accelerated to ~400 kHz
(d) SPI transfer speed was doubled when initializing the SDfat.h library.

The RTC triggered reading + buffering event occurs 64 times before the 4 kB EEPROM is full and the SD data save is performed. This sequence can be considered the minimum run-time duty cycle. Comparison of the amount of power required to complete one run-time cycle, to the power consumed while the logger is sleeping, helps determine when the run-time power consumption becomes a significant factor in the operational lifespan of the data logger. If this drip counter sleeps at 0.25 mA, then the battery has to supply 21,600 mAs per day even if the logger does not wake up. If that same logger requires 5.07 mAs per run-time cycle (Table 3), it would have to execute 4320 of those minimum run-time cycles to consume 21,600 mAs. 

Reaching the point where run-time and sleep-time power requirements are equivalent would require the logger to buffer more than 3 distinct data records into the EEPROM per second. However, the optimized SD save event takes 433 ms (Figure 9c). This prevents more than two records from being recorded per second no matter how much power is available. Adding a safety margin for random SD latencies and slow sensors, suggests 1 Hz as the practical upper limit for discrete timestamped records with the Cave Pearl logger design. For the logger in this example, this is ~1/3 of rate needed for run-time events to equal the equal the power consumed while sleeping. A 1 Hz record-saving rate would therefore reduce this logger’s operating lifespan by ~30%.

The DS3231 RTC used on this logger supports I2C bus clock frequencies up to 400 kHz but this is four times faster than the nominal 100 kHz specification of the AT24C32 EEPROM included on the RTC module. This accelerated bus speed has not resulted in data loss over multiple deployments and most sensors support frequencies higher than a 3.3 V Pro Mini style Arduino’s 400 k Hz maximum.

**Figure 10:**
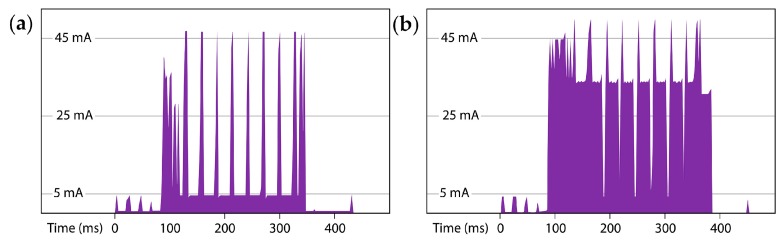
Current draw from a 4.6 V supply by different SD cards accessed in SPI mode: (**a**) Current drawn during a power optimized save to a no-name 1 GB microSD card that ***is*** compatible with 512 byte data saves. (**b**) Current draw on that same logger running the same code using a SanDisk 2 GB microSD card that is not compatible with small blocks sent over the SPI interface. Though the data was recorded without error, the event required 10.33 mAs which is ~4 times the power consumption of the 1 GB card. These records were captured with an Arduino UNO measuring the voltage drop across a 12 Ω shunt resistor at ~89 kHz (ADC prescalar = 8) [37].

Any chip-based device can exhibit unexpected power consuming behaviors and SD cards provide a particularly relevant example. Modern microSD cards are designed to be used on a high-speed Secure Digital Input Output (SDIO) bus with 16 kB (or larger) multi-block writes and reads. Large capacity cards must emulate the slower 512 byte block-access that Arduino libraries use, leading to the erasing and rewriting of flash memory areas much larger than the data actually being saved (Figure 10). Nokia SD cards in the 128 to 512 MB size range do not suffer from this problem and generally consume 50% less power than other branded cards for identical data saving events. Older 256 and 512 MB SanDisk brand cards also perform well with the Cave Pearl data logger, delivering low sleep currents between 60–80 µA [36]. SD card sleep currents can be checked with an ammeter during breadboard testing.

### 6.4. Power Considerations: Conclusion

The software-side techniques described in this section have already been embedded in the drip logger example code provided on GitHub [22]. The hardware based strategies such as SD card testing, or RTC pin powering, have to be implemented at the time of the build. Hardware methods *are not required for successful logger operation*, although they can extend the operational life to achieve multi-year deployments.

Loggers built to the basic plan, with no RTC pin powering (Figure 1), typically sleep at ~0.20 mA before sensors are added, easily reaching twelve months of operation using 3 AA cells in series as the main power supply (Figure 6). The maximum dropout voltage for the MIC5205 regulator commonly used on mini-style Arduino boards is 350 mV. To protect SD card data, a data logger should go into shutdown mode when the battery pack reaches: 3.65 V (i.e., the regulator’s 3.3 V output plus the maximum dropout voltage under load). At that point, each of the individual cells in a 3 battery pack is still at ~1.20 V, leaving up to a third of their capacity unused. Experience has shown that drawing alkaline batteries down to the 0.8 V level quoted in performance specifications significantly increases the probability of battery leakage, especially when field logistics prevent retrieving the units on schedule.

Longer operational lifespans can be achieved by adding parallel battery banks which are isolated from each other with 1N5817 Schottky diodes. Power supplies with up to 4 banks of the 3 × AA batteries have been successfully deployed on Cave Pearl units driving high-drain sensor combinations. Even with that extra capacity, it is recommended that field deployments use primary cells with anti-leak protection.

## 7. Case Study 1—Monitoring Vadose Zone Hydrology Using Drip Counters

### 7.1. Nature of the Problem

Cave speleothems are secondary calcite deposits formed in cave voids by infiltrating water and they provide valuable multi-proxy climate records with global distribution [38]. Understanding how the climate signal is transmitted and modified as meteoric water passes through the unsaturated vadose zone is an area of active research, with the goal of improving paleoclimate reconstructions from speleothem calcite.

Cave drip rates have been studied in the past with manual counts/collections. That data was only available during site visits, or as averages over the time between visits if water was collected. Some sites were instrumented with surface meteorological equipment designed for relatively large fluxes. Scale mismatch and large power requirements spurred the development of custom monitoring solutions [39], with new instruments like the ~£300 “Stalagmate” drip counter by Driptych providing quality field data for a number of drip monitoring projects [40,41,42]. An inexpensive open-source alternative, suitable for use in caves with a range of sensors would facilitate larger installations.

### 7.2. Drip Counter Construction

The drip counter housing is composed of a flexible PVC end cap (such as the “Fernco Qwikcap”) connected to a 4 inch diameter rigid PVC drain cap with a stainless steel pipe clamp (Figure 5a,b). The sensor is an ADXL345 accelerometer operating in tap-sensing mode and the impact of a drip on the surface of the housing sends an alarm-low signal to the Arduino (on pin D3). This triggers an interrupt subroutine which increments a counter variable before returning the logger to sleep. At a user defined interval, the RTC alarm (connected to pin D2) wakes the logger, which then saves the accumulated drip-count to the EEPROM buffer, with a timestamp and a reading from the temperature register in the RTC. Accelerometer axis readings are recorded once a day to confirm that the unit has not changed position during the deployment. 

### 7.3. Field Deployment

**Figure 11:**
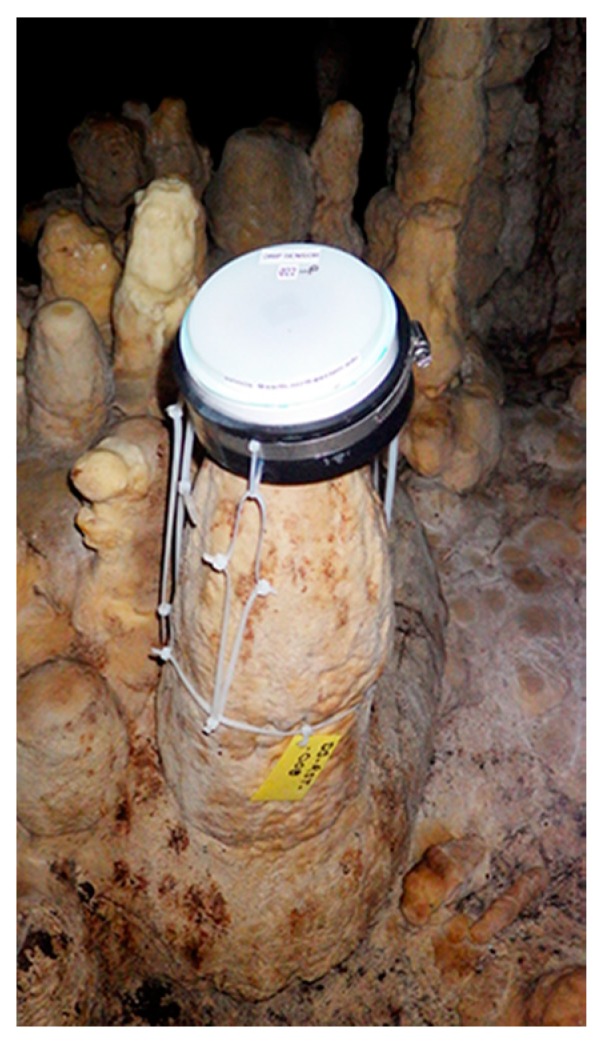
A drip-sensor tethered by cable ties to the top of a stalagmite, at a slight incline to prevent water accumulating on the surface.

In September 2014, six prototype drip counters were deployed in the Rio Secreto section of the Sistema Pool Tunich cave, located ~5 km from the Caribbean coast of the Yucatan Peninsula, Mexico. This increased to 20 units by the end of 2015, distributed in clusters of 3–4 units in 4 chambers of the cave. Drip counters are harnessed to the tops of stalagmites using cable ties, at a slight angle to prevent accumulated water from affecting the count (Figure 11). Six service trips have been completed in the 36 months since the initial deployment and the cumulative total is greater than 450 months of drip logger operating time. Additional Cave Pearl loggers were deployed with other sensor combinations to provide correlated data on rainfall, temperature, barometric pressure, relative humidity and water level.

### 7.4. Performance and Validation

Six drip counter units have failed to date. The majority of these failed in the first year of deployment with the accelerometer set to its maximum sensitivity. This caused some units to double- count or to enter self-triggering loops which rapidly depleted the batteries. Deployments from 2015 to present show that reduced sensitivity on the ADXL345 still allows for reliable drip detection over fall distances between 0.2 to 8 m. The drip counting event uses so little power that loggers which sleep below 0.25 mA will run >1 year on three alkaline batteries despite drip count variations over two orders of magnitude.

The housings have proven to be robust, with no significant water ingress despite the upward facing seam of the flexible cap on the housing. Desiccant indicator beads inside the unit do begin to shift color on deployments longer than 6 months, possibly due to slow water-vapor permeability at the solvent welded seam. The project now uses two 10 g pouches of silica gel desiccant inside the unit for deployments lasting more than one year. 

**Figure 12:**
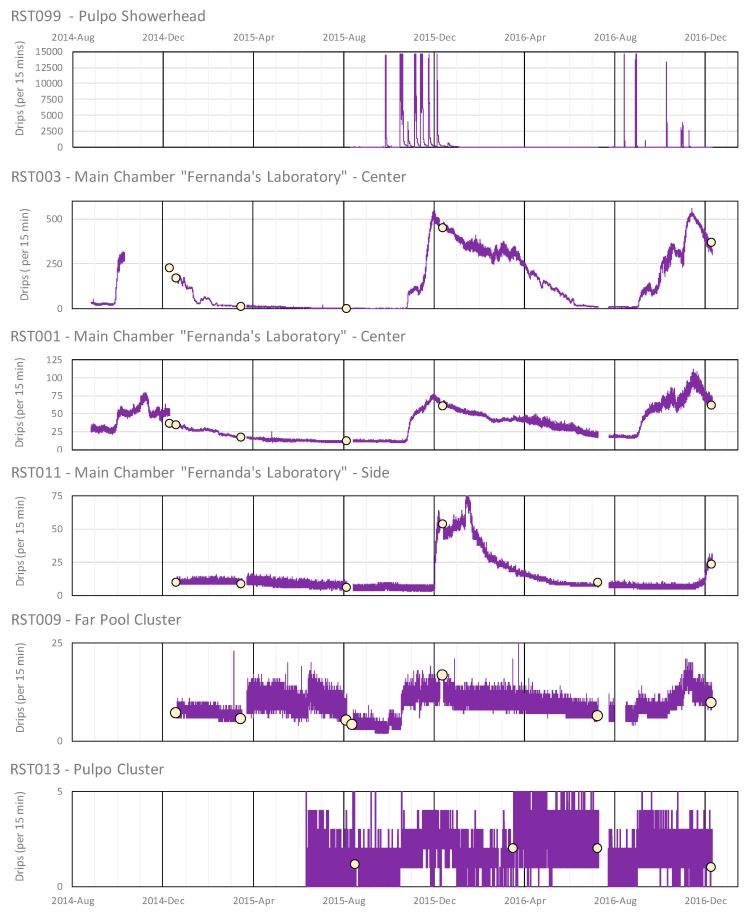
Drip data from Cave Pearl loggers (purple line) with manual drip counts (yellow circles) in units of drips/15 min from long-term monitoring stations in the Rio Secreto section of the Pool Tunich Cave System. Panels are arranged from high to low drip rates, with the top panel showing the Pulpo Showerhead that episodically exceeds the instrumental limit of ~15,000 drips/15 min and the bottom panel showing RST013 with less than 5 drips/15 min.

**Figure 13:**
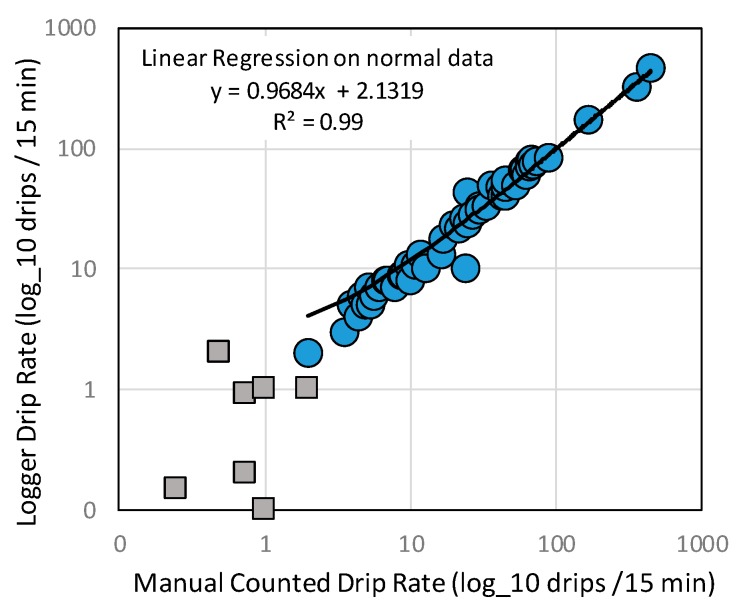
Correlation between manually counted drip rate (shown as drips/15 min on *X*-axis) against number instrument counted drips (x/15 min on *Y*-axis). Linear regression on normal data has an R^2^ of 0.99, excluding counts where either manual or instrument reading is 1 drip or less/15 min (squares). Both axes shown as log_10.

The accuracy of the drip counters is demonstrated by comparison of manual counts with the logger data (Figure 12). The R^2^ of manual counts compared to logger records is 0.99 (Figure 13), excluding observations where either manual or instrument count was below 1 drip/15 min. Maximum instrument response is ~15,000 drips in 15 min and this was exceeded at station RST099–Pulpo Showerhead (Figure 12a). The Pulpo Showerhead is a shaft created by penetrating tree roots that have since rotted away. That drip point has been dry during every service-visit but the 2 m diameter calcite mound beneath the shaft corroborates the recorded data showing episodic flows associated with large recharge events. Even when flow rates exceed the units drip counting limit, the data does capture the duration of the event. 

### 7.5. Discussion

Flooding events knocked some of the units off-station during the early deployments but the displaced units yielded long strings of zeros in the record, indicating that internal sensor noise was not creating false-positive counts in the absence of drip impacts. Units that were not displaced by the flooding recovered gracefully after months of submersion with no apparent hysteresis.

The maximum count per interval from the drip-sensor configuration is constrained by ~75 ms of added processor sleep before the tap sensing alarm can be reactivated. This delay was required to allow the housing surface to stop vibrating and in the case of drip falls greater than 8 m, even longer delays were required to prevent double counts. Indicator LED blinks during sensor/interrupt events made it easy to spot and correct these over-counting situations. While this case study describes a novel application of tap-sensing functionality, having to wait for the vibrations to die down is a fundamental limitation of the accelerometer based method. These added delays cause a corresponding reduction in the temporal resolution but the logger itself can process interrupts several orders of magnitude faster than drip sensing requires. The design can be repurposed for sensors generating frequency pulses into the 50 kHz range, depending on how much code is executed in the interrupt service routine.

## 8. Case Study 2—Tracking Water Flow in a Flooded Cave System

### 8.1. Nature of the Problem

The Yucatan Peninsula of Mexico is experiencing rapid urbanization and tourist development on a coastal karst aquifer which serves as the only source of potable water for the region. This aquifer includes an extensive network of flooded conduits of 10–100 m in width [43] with >1300 km surveyed to date. The aquifer is density stratified, with a mass of fresh meteoric water floating on top of intruding marine waters. The aquifer is broadly influenced by the coastal semi-diurnal micro tides (~30 cm amplitude), with an annual range of ~70 cm. Reversing and bi-directional fresh-saline flows are common [44]. Aquifer discharges include submarine springs or blue holes and rocky sided estuaries (locally called caletas) formed where the cave ceiling at the coastline has collapsed [43]. Technical SCUBA diving is the method of choice for accessing these subterranean estuaries.

Large oceanographic flow meters have been used in several locales to obtain time series of point velocity in flooded karst conduits (Yucatan [45]; Bahamas [46]; Florida [47,48]). These instruments were not optimal for subterranean use, as their physical size and mass require lift-bag deployments and anchoring chains. Even with smaller units now available, the cost of $5000–10,000 per unit is still prohibitive for a large network of sensors. 

### 8.2. Flow Meter Construction

Simple pendulums can be used to infer velocity by measuring the tilt angle of a body suspended in a flowing liquid [49] and several research groups have developed open water instruments for coastal deployments based on the principle. Examples include the commercially available Lowell TCM-1 [50], a buoyant tethered sphere [51], the URSKI float design [52] and a load-cell based variation [12].

**Figure 14:**
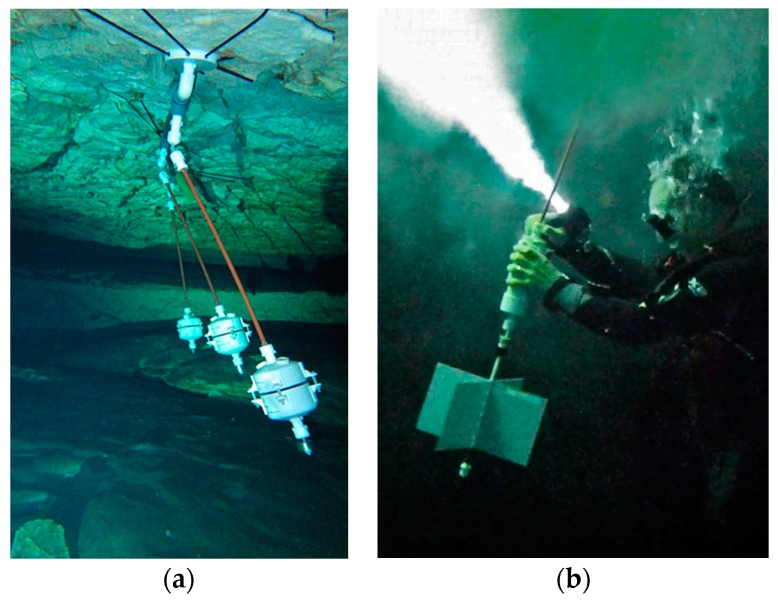
Deployments of tilt flow meter: (**a**) An early flow meter installation in the Casa Cenote cave, at ~300 m from the blue hole discharge. Multi-logger deployments with ~1 m spacing allow assessment of inter-unit variability. Flow sensors are ballasted to slightly negative buoyancy, allowing soft bungee cord anchors that cause no damage to the cave. (**b**) High surface-area flags in low-flow conditions mechanically enhance the instrument response.

This project is currently using an LSM303DLHC accelerometer to measure tilt angle and bearing is determined from the magnetometer (compass) present on the same IC chip [53]. The sensor module is mounted inside the lid of the submersible housing using double sided tape (Figure 5c). Early body prototypes used a housing constructed from 4 inch PVC end caps suspended from a moving rod (Figure 14). A low friction pivot joint fashioned from cable ties with mounting holes allows the assembly to swing freely.

Each accelerometer is calibrated by taking measurements while slowly spinning the fully assembled logger through orthogonal 360° rotations [54]. Those readings are processed with Magneto v1.2 software [55] to determine correction factors for the sensor’s internal offset and sensitivity errors. The units are then suspended from a pivot for several hours to quantify offsets created by the physical mounting. Noise in the sensor readings is reduced with a smoothing filter which disposes of the two highest and the two lowest values from a 13 reading burst sample, captured every 15 min [56]. The final angle of inclination is calculated from the averages of those remaining raw axis readings.

### 8.3. Field Deployment

Cave Pearl flow sensors were installed beginning in December 2013 in the flooded cave discharging at the coastal site of Casa Cenote, which was previously the site of an Aanderaa RCM 7 flow meter installation from February 2000 through to November 2001 [45]. 

The flow meters are suspended from anchors plates tied to the ceiling of the flooded cave. Five gram stainless steel washers were added to the bottom of the housing until the rod and logger combination have slight negative buoyancy. Since rod length does not affect the tilt angle, the rods were sized to place the logger bodies at the vertical center of the passage. A combined pressure and temperature sensor was co-deployed on the ceiling nearby to monitor tide levels.

### 8.4. Performance and Validation

Several units suffered power failures in 2014 due to epoxy degradation, with salt water shorting sensor boards on the outer surface of the housings. No similar problems have occurred with Loctite E30CL epoxy. The 316 stainless steel washers used for ballast corrode significantly over deployments greater than six months but no biofouling has occurred in the low energy cave environment. 

Significant vortex shedding was observed with early prototypes. This was reduced on subsequent deployments by increasing the internal mass of the units with a second bank of AA batteries and by adding a 120 ms time delay between the raw sensor readings before they were processed by the smoothing function. The change from the larger 4 inch round body (Figure 14) to smaller 2 inch diameter cylindrical bodies (Figure 5e) also helped reduce vortex shedding.

**Figure 15:**
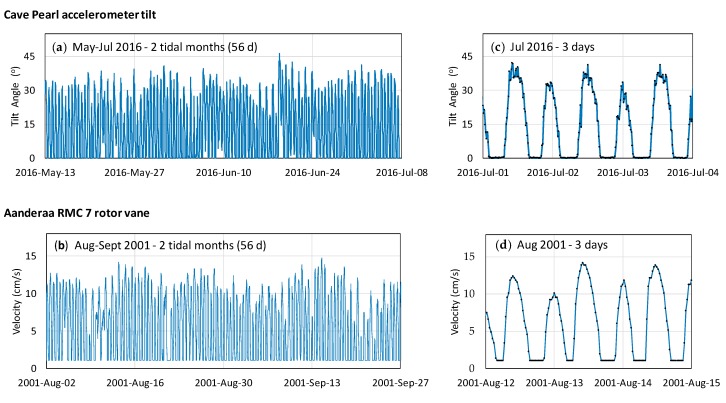
Casa Cenote flow meter data comparison between Cave Pearl accelerometer-tilt flow meter (upper row) and Aanderaa RCM 7 rotor vane system (lower row). The data over 2 tidal months (56 days) shown in left panels (**a**,**b**) for representative periods without events and over 3 days in right hand panels (**c**,**d**).

The tilt flow meters respond well to the full range of water flow at the site (Figure 15a). The data series show clear semi-diurnal fluctuations from zero tilt during high tide slack-water periods to proportionally increased tilt angles during high-flow periods caused by sea level and spring-neap tidal cycles. The qualitative character of the data is highly comparable to that of an Aanderaa RCM 7 over 56 days full tide months (Figure 15a,b) and also at a finer daily scale (Figure 15c,d).

The Aanderaa RCM 7 data appears smoother as the unit accumulates individual rotor counts every 36 s and then stores a 30 min averaged value [57]. In comparison, the accelerometer tilt angles are calculated from only 13 values taken over 1.5 s at 15 min intervals, so that data is affected by turbulent eddies and vortex shedding during high flow periods. Both data sets show the slight “left” skew on the velocity (or tilt) curves (Figure 15c,d), which is tied to hysteresis in the conduit flow, with slower decline in discharge during ebbing tides. The minimum recordable velocity for the Aanderaa RCM7 is 1.1 cm/s and the flat-line minima seen in each tide cycle was originally thought to be an artifact of the heavy gimble-mounted unit being incapable of rotating in response to reversing pipe-flows. The freely-hanging hydrometric pendulum method shows this to be an interpretation error and the Cave Pearl data unequivocally demonstrates the regular occurrence of slack water. 

### 8.5. Discussion

The tilt-flow meter configuration provides detailed records from challenging sites such as the Casa Cenote subterranean karst estuary, which is only accessible using technical cave diving. The cylindrical-body flow meters have been on deployment more than 2 years at a range of depths. Now that the underwater housing has passed this real world test, it is appropriate to begin point velocity calibration in a hydrological flue, or by co-deployment with an acoustic Doppler current profiler. This will necessarily be an empirical calibration, as the irregular body profile varies with tilt angle, leading to a strongly non-linear response. 

Buoyancy control is critical for data consistency with this method but this has proved challenging. Even when the housings are identical, AA batteries from different manufacturers (and different batches from the same vendor) vary in weight by more than 10%. Corrosion causes mass loss from the “marine grade” stainless steel ballast washers and some coastal sites range from 10–100% marine over each tidal cycle due to ocean water intrusion. Each unit is trimmed to 5–10 g negative at the time of deployment, so that some loss of the ballast still leaves the unit slightly negative.

Deployments at sites with very low flows showed displacements barely above accelerometer noise. Attempts to address this mechanically by attaching large surface area ‘drag flags’ to the bottom of the housings successfully increased the response by a factor of four (Figure 12b). Unfortunately, these flags generate lift when they pass ~45° of inclination. This significantly distorts the instrument response and making the approach unsuitable for sites which also experience high-flow periods.

The underwater housing fits safely in a mesh equipment bag and has proven to be easy for technical divers to deploy and exchange. This kind of flow detector is well suited to exploratory deployments, providing an inexpensive way to compare the qualitative hydraulic behavior and responses between numerous sites. This case study also illustrates how DIY sensor development inevitably requires calibration protocols which can be achieved with a similar level of resources. Calibrating unique instruments can easily require more time and effort than building or programming. However, even without that level of accuracy, simple devices like the Cave Pearl flow meter could be used to gather information that would enable more effective deployment of expensive commercial instruments in complex systems.

## 9. Conclusions

The Cave Pearl Project provides a simple three-component data logging system constructed from inexpensive and interchangeable components. The logger supports sensors suitable for a wide range of environmental monitoring applications and the flexible connection plan makes it easy to re-arrange the core components into different physical configurations. Power optimization focuses principally on reducing sleep current, leading to operational lifespans exceeding a year on AA batteries. Techniques for optimizing run-time current are also presented, which become important for projects that require high frequency sampling. While sensor input can be captured on the scale of kHz, this platform can save discrete time-stamped records at maximum rate of ~1 Hz.

The two environmental housings are constructed with commonly available PVC fittings using basic shop tools. The surface housing withstands shallow submersion, or extended periods in 100% relative humidity. The submersible housing has a sliding-cap design which increases the strength of the O-ring seal at depth and units have been deployed at depths up to 30 m for >3 years without water ingress. The two case studies illustrate how these DIY housings enable similar sensor configurations to monitor dramatically different aspects of a groundwater system.

These case studies present only two of the many sensor configurations currently being used in a mini “Critical Zone Observatory” with 50+ loggers in the Yucatan Peninsula. Using I2C sensor breakout boards from the hobbyist market, Cave Pearl loggers are monitoring meteorological inputs, surface processes, density-stratified groundwater flows and coastal discharge on the imperiled Meso-American barrier reef. 

Other complex environmental systems can be similarly instrumented with this logger to observe whole-system responses to changing boundary conditions. At less than $50 per unit, modest research budgets can sustain a substantial number of these devices and create custom sensor arrangements for research questions that are not well served by existing commercial solutions. Open source prototyping platforms enable people with little electronics or programming background to build instruments capable of collecting primary data and it is hoped that the release of the Cave Pearl modular data logging system supports this endeavor in both research and educational settings. 

## Figures and Tables

**Table 1:** Prioritized list of techniques used for optimizing sleep current on Cave Pearl data loggers and associated sleep current reduction.

Techniques for Optimizing Sleep Current	Approximate Reduction
Put the microcontroller into the deepest power down modes between readings	5 mA
Disconnect ‘always-on’ indicator LED’s on the Arduino and on sensor boards	~5 mA each
Choose 3.3 V sensor IC’s with low-power sleep modes	0.05–0.1 mA each
Power the RTC from an Arduino output pin, rather than the 3.3 V rail	0.09 mA
Remove power from sensors/devices via a transistor controlled by a digital pin	sensor dependent
Test and select SD cards with the low sleep current	0.05–0.1 mA
Select an Arduino that uses a more efficient MCP1700 series regulator	20–25% savings
*When combined, these techniques can reduce a data logger’s sleep current to ~0.1 mA.*	

**Table 2:** Prioritized list of techniques used for optimizing run-time current on Cave Pearl data loggers and associated current reduction.

Techniques for Optimizing Run-Time Current	Approximate Reduction
Put the processor to sleep while waiting for sensor readings	5 mA × Δt
Use higher bus speeds to accelerate sensor communication	5 mA × Δt
Select sensors that use less power while taking a new reading	Up to 5 mA × Δt
Use green indicator LEDs with large limit resistors	2 to 5 mA × Δt
Disable on-chip peripherals with Power Reduction Register settings	0.01–1.0 mA × Δt
*When combined, these techniques can reduce average run-time currents by up to 90%*	

**Table 3:** Total charge and duration of the duty-cycle events shown in Figure 9.

	Drip	RTC	SD Save
**Before Optimization:**			
Duration (ms)	87	140	1200
Charge required (mAs)	0.2324	0.4056	5.980
**After Optimization:**			
Duration (ms)	89	157	433
Charge required (mAs)	0.0050	0.0403	2.496

## Appendix A

**Table A1:** Components and price list for cave Pearl data logging platform and the surface (drip sensor; Case Study 1) and submersible (tilt flow meter; Case Study 2) environmental housings. The 2018 prices are based on delivery to Midwest USA. Items without a suggested supplier are generally available at electronics retailers or hardware stores.

Component	Source	Cost/Unit
**Data Logging Platform**		
Generic 3.3v 8MHz Pro Mini clone [328P]	eBay	$2.00
DS3231 & AT24C32 I2C RTC Module	eBay	$1.25
CR2032 RTC battery	eBay	$0.50
(2) 12mm M2 nylon standoffs for RTC	various	$0.40
microSD card adapter for Raspberry Pi	eBay	$1.00
128-256Mb microSD (Nokia, Sandisk) (used)	eBay	$3.00
3xAA series, or 3 single AA plastic battery holders	eBay	$1.00
Deans T-Style Battery Connector	eBay	$1.00
4 inch or 2 inch Oatey Test Cap	Menards [#39103/#6893208]	$0.50
(2) WSD1241 Micro 4B Plug	Advantage Hobby	$3.00
Misc parts: wire, caps, resistors, LED, etc	various	$1.50
	**Total in US Dollars:**	**$15.15**
**Drip Sensor Housing (Case study #1)**		
FERNCO QWIK Cap, 4 inch	Menards [#QC-104]	$3.90
4 inch PVC Drain Cap	Zoro Tools [#G6182477]	$1.25
Charlotte 4-in dia PVC Test Cap	Lowes [#23408]	$2.05
ADXL345 accelerometer module (w 3.3v input)	eBay	$2.00
	**Total in US Dollars:**	**$9.20**
**Flow Sensor Housing (Case Study #2)**		
(2) 2 inch Formufit white table caps	Home depot [#F002ECT-WH-4]	$4.50
(1) Formufit End Cap, 2 inch Slip fit	Home depot [#F002EEC-WH-10]	$1.60
(6 inches) Formufit 2 inch Sch. 40 PVC pipe	Home depot [#P002FGP-WH-5]	$1.50
2 inch Schedule 40 PVC slip fit coupling	Menards [#K00955T]	$0.50
(2) 1/2 inch NPT Threaded PVC Plug Sch. 80	Zoro Tools [#G2988937]	$2.80
332 O-Ring, EPDM, 3/16 dia.	Zoro Tools [G2506561]	$1.00
(3) 3-1/2 inch, 1/4-20, Nylon 6/6 Hex Bolt, Slotted	Amazon	$1.00
OR: Threaded Rod 1/4-20 x 4 ft, Nylon	Zoro Tools [#G0540757]	
(3) Black 6/6 Nylon Hex nut, 1/4-20	Zoro Tools [#G2205567]	$0.25
Epoxy, Loctite Hysol 30-CL, 50mL	Zoro Tools [#G2233901]	$1.50
GY-511 LSM303DLHC Module	eBay	$3.00
	**Total in US Dollars:**	**$17.65**
